# A cascaded U-nets model based on hybrid attention for video anomaly detection

**DOI:** 10.1371/journal.pone.0357967

**Published:** 2026-10-29

**Authors:** Suyuan Li, Nan Jiang, Jia Qin, Jiangchang Gong, Yongheng Pang, Fuyao Wang

**Affiliations:** School of Public Security Information Technology and Intelligence, Criminal Investigation Police University of China, Shenyang, China; PLOS, UNITED KINGDOM OF GREAT BRITAIN AND NORTHERN IRELAND

## Abstract

The aim of video anomaly detection is to identify events that deviate significantly from normal behavior. The majority of the current methods for identifying anomalous behaviors rely primarily on frame reconstruction and frame prediction. However, due to the powerful representation and generation of deep neural networks, it is not sufficient to assume that anomaly events in the reconstruction methods have larger reconstruction errors. Likewise, prediction methods are not robust to noise, which can impact the effectiveness of anomaly detection. In this paper, we propose an anomaly detection method based on cascaded attention U-Nets, combining the advantages and balancing the disadvantages of the reconstruction methods and the prediction methods. Specifically, by integrating the spatial attention module and transformer-based channel attention module into the cascaded U-Nets, the weights of the spatial regions and the channel regions that represent background features can be decreased. Furthermore, to fully utilize the temporal information, a temporal shift module is incorporated into the prediction network to improve future frame prediction. Extensive experiments were conducted on several public benchmark datasets, such as the UCSD Ped dataset, the CUHK Avenue dataset, the ShanghaiTech dataset and the UCF-crime dataset. The results indicate that the proposed method can effectively enlarge the gaps between the normal events and the anomaly events, and achieve AUC 95.6% on the UCSD Ped2 dataset, 84.1% on the UCSD Ped1 dataset, 87.6% on the CUHK Avenue dataset, 75.1% on the ShanghaiTech dataset, 73.5% on the UCF-crime dataset, which outperform other previous state-of-the-art methods.

## 1. Introduction

Since cameras have been so extensively used and quickly developed, numerous individuals leverage video surveillance to increase public safety [1]. Generally, the need to analyze an enormous amount of video data in real time can result in the waste of resources necessary for human verification. Video anomaly detection is the timely detection of a small number of anomalies that are incongruous with the majority of normal incidents using computer vision, which can save labor costs and improve the effectiveness of video monitoring. Despite receiving a lot of attention throughout an extended period of research, it is still extremely challenging to determine anomalies in the actual world. The primary challenges that occur are as follows: (1) It may prove challenging to create boundaries that clearly define anomalies, which often rely on the related context. For example, running by people is seldom seen in public areas like malls and subway stations but is common in parks. (2) It is usually challenging to gather enough samples and labels to model each kind of anomaly since anomalies have a lower likelihood of occurring than normal events.

Unsupervised learning methods with low demand in the training set are implemented to identify anomalies in videos so as to address the aforementioned problem. To train an anomaly detection model to acquire a general distribution of normal activities, only normal data are employed. Outlier samples are those that obviously deviate from the normal distribution during the test phase. Generally, existing anomaly detection methods can be broadly classified into two groups based on deep-learned features and classic hand-crafted features. In the classic methods based on hand-crafted features, model creation and feature extraction are the two key components. First, low-level spatial-temporal information can be collected, including histograms of optical flow, histograms of oriented gradients, and so on. Then, to encode normal patterns, statistical models, sparse coding models, and dictionary learning are implemented. However, the performance of the model will be limited by the lengthy and unstable quality of the feature extraction and model construction processes used in these classic methods.

Recently, deep-learned features [2,3] have surpassed hand-crafted features in anomaly detection since the invention of deep neural networks, including Recurrent Neural Networks (RNNs) [4], Convolutional Neural Networks (CNNs) [5], Generative Adversarial Networks (GANs) [6], and Transformers [7]. In detail, reconstruction-based methods and prediction-based methods are two broad categories that apply to methods based on deep-learned features. Reconstruction-based methods, like Auto-encoder [8], exclusively are trained on normal samples in an effort to reconstruct them with smaller errors. Larger reconstruction errors within an instance will be deemed an anomaly. Yet, it is possible to correctly reconstruct some anomalous incidents, which commonly leads to missed detection. In this case, since deep neural networks have tremendous representational and generational capabilities, it is not sufficient to assume that anomalous occurrences have greater reconstruction errors. Consequently, the frame prediction model is constructed in anomaly detection to mitigate the limit of the reconstruction error. By predicting the next future frame using several previous continuous frames, the gaps between normal and anomaly frames can be extended. This is attributed to the fact that unexpected appearance and motion compared with normal events cannot be predicted well, further resulting in larger prediction errors between predicted frames and ground-truth frames for anomaly events. However, prediction methods mainly focus on the variation of appearance and motion, which is sensitive to any changes in these frames, resulting that the normal events may gain a larger predicted error.

In order to ameliorate the challenges mentioned above, a hybrid framework based on frame prediction and frame reconstruction is constructed, taking into account the advantages and disadvantages of reconstruction-based methods and prediction-based methods. Particularly, the frame prediction model can boost anomaly prediction errors, strengthening the ability to identify anomalous events. Similarly, the frame reconstruction model can improve the quality of predicted normal frames and increase their tolerance to noise and other challenges. Moreover, the capacity to detect anomalies depends on temporal correlation, which is critical for prediction models. In general, 3D CNN can be integrated with prediction models to achieve good performance with high computational cost. Likewise, optical flow can be considered as a constraint on motion between continuous frames to express temporal correlation. Nevertheless, factors like occlusion, light illumination, and other things can affect the optical flow, which will further influence the effectiveness of anomaly detection. Therefore, a general and efficient temporal shift module (TSM) is inserted into our prediction model to focus on temporal correction. In such a manner, the reconstruction-prediction errors of anomaly frames can be enlarged, and those of normal frames can be reduced. The following is a list of our contributions:

(1) We integrate frame prediction and frame reconstruction networks for anomaly detection, which can effectively enlarge the gaps between the normal frames and the anomaly frames.
(2) We design cascaded U-Nets with spatial attention module and transformer-based channel attention module, the first U-Net is utilized as a frame prediction network, while the second U-Net is employed as a frame reconstruction network.
(3) We leverage the temporal shift module to model temporal information in the prediction network, which can enhance the quality of the predicted frame.
(4) We propose the fusion of prediction score and reconstruction score to distinguish the anomaly event, which can improve generalization ability. The performance of the proposed method can be concluded through experiments on three public datasets. The results demonstrate that the proposed method can surpass other state-of-the-art methods.

The remainder of this paper begins with related works of video anomaly detection in Section 2. Following that, Section 3 introduces the proposed cascaded attention U-Nets framework, which consists of the spatial attention module, the temporal shift module, transformer-based channel attention module, the loss function, and the anomaly score. The results of the experiment on three public datasets are provided in Section 4. Finally, in Section 5, a conclusion is given.

## 2. Related works

During the past few years, the detection of video anomaly has grown as a hot research topic in computer vision. All currently used methods can be broadly categorized into two groups: methods based on classic hand-crafted features and methods based on deep-learned features. Next, the most relevant anomaly detection methods will next be reviewed in more detail.

### 2.1 Classic anomaly detection based on hand-crafted features

Traditionally, visual hand-crafted features [9,10] are incorporated into classic approaches to represent each video sequence. Specifically, these classic anomaly detection methods based on hand-crafted features can be roughly into distanced-based methods and probabilistic-based methods. To implement distance-based methods, training data can be used to construct a model of normality that assesses the similarity of video frames to one another. Anomaly scores are then determined by measuring deviations from the model. Saligrama et al. [11] propose a fundamental nearest neighbor location-dependent anomaly detection approach, which adopts flow fields or blurred foreground masks as hand-crafted video representations, and uses normalized L1 or L2 voxel-wise distances as hand-crafted distance measurements. In [12], by aggregating the K-nearest neighbors (KNN) distances between the normal and test video, the anomaly scores can be obtained for overlapping fixed-size video volumes, which are able to be represented as low-level motion descriptors. Lu et al. [13] propose a novel distance-based video anomaly detection, locality-sensitive hashing (LSH) is utilized to map the samples whose similarity surpasses a particular threshold into the same bucket in advance. Different from distanced-based methods, by training the model, probabilistic-based methods are able to learn the probability distribution of normal behaviors in video data. The probability value generated by the test sample is then compared with the threshold to further determine if a certain behavior is anomalous. Usually, probability methods that are often employed involve the Social Force Model [14], Hidden Markov Model [15], Markov Random Field [16], Gaussian Mixture Model [17], and others.

### 2.2 Anomaly detection based on deep features

Convolutional neural networks (CNNs) have achieved significant advancements in a variety of computer vision applications, including action recognition and objection detection [18–22]. In previous years, video anomaly detection methods based on deep-learned features have shown success. Generally, these anomaly detection methods based on deep-learned features can be approximately divided into reconstruction-based methods, prediction-based methods and hybrid methods.

#### (1) Reconstruction-based methods.

These methods generally employ auto-encoders and variational auto-encoders to reconstruct the input frames and learn the distribution of the normal events. Anomaly events can then be identified according to the notion that the anomalies cannot be precisely reconstructed in terms of the above distribution. In [23], an end-to-end fully convolutional feed-forward auto-encoder learning architecture is constructed to train both the local features and the classifiers. In order to better handle the uncertainty gained by insufficient data sources, Huang et al. [24] present a conditional variational auto-encoder architecture with Margin learning embedding and variational normal inference modules. Gong et al. [25] propose a memory-augmented auto-encoder, where normal data can be represented by encouraging and updating the memory contents, enlarging the reconstruction error to better identify anomalies. In [26], an incremental spatiotemporal learner coupled with an auto-encoder is used to continuously update and distinguish between anomaly and normality that alter over time. Different from the mentioned methods, some two-stream models are employed to capture the appearance and motion for decisions that are more precise. In [27], optical flow patches and the gradient are, respectively, taken into consideration as inputs for each stream in the two-stream architecture based on a multivariate Gaussian fully convolution adversarial auto-encoder. In order to improve detection performance, Chang et al. [28] develop a novel convolutional auto-encoder architecture that can simultaneously capture both spatial and temporal information. In [29], a dual generator architecture with an influence-aware attention module has the capability of quantifying the interactions in the scene from both a spatial and a temporal perspective. Furthermore, some sequential data models are widely used in anomaly detection in order to completely account for the time dependency. Luo et al. [30] propose ConvLSTM-AE framework, CNN and ConvLSTM are respectively leveraged to focus on appearance and memorize motion information, better encoding the change of appearance and motion for normal events. Zhao et al. [31] utilize 3D convolution and 3D de-convolution in the encoder and decoder, respectively, improving the capacity to extract motion patterns from temporal dimensions for identifying abnormalities. Sun et al. [32] propose a hierarchical semantic contrast (HSC) method for scene-aware video anomaly detection, leveraging pre-trained models for semantic feature extraction, combining scene-level and object-level contrastive learning within an autoencoder framework, and using skeleton-based motion augmentation to enhance rare normal activity learning. In [33], Dong et al. propose a novel reconstruction-based video anomaly detection with idempotent generative network, which is composed of the novel reconstruction module namely PSVAE and an idempotent loss term. However, in reconstruction-based methods, since deep neural networks have tremendous representational and generational capabilities, it is not sufficient to assume that anomalous occurrences have greater reconstruction errors. Although reconstruction-based methods have been shown to be effective in video anomaly detection, relying solely on frame reconstruction has inherent limitations and the generalization ability of the model is weak.

#### (2) Prediction-based methods.

With the development of Generative Adversarial Networks (GANs), video frame prediction has received a great deal of attention recently since it offers so much potential. In prediction-based methods, the next frame can be predicted by several given consecutive input frames. Based on the assumption that the motion and appearance of anomaly events are unpredictable, the events with larger prediction errors will be considered as anomalies. For instance, Liu et al. [34] are the first to leverage the difference between a predicted future frame and its ground truth to detect an abnormal event, while appearance constraint and motion constraint are applied to predict a future frame with higher quality for normal events. To avoid ignoring the reverse time information, Zhong et al. [35] provide a bidirectional spatio-temporal feature learning framework that incorporates forward and backward features from double-encoder networks for bidirectional prediction. In order to prevent the overgeneralization of auto-encoders for anomaly detection and expand the gaps between normal frames and anomaly frames by lowering the prediction errors for normal frames, Zhang et al. [36] employ a hybrid attention and motion constraint in the auto-encoder. To address the foreground-background imbalance issue, Zhou et al. [37] present an anomaly detection approach based on the data-dependent attention-driven loss. A single mask map can be calculated to pay more attention to sparse foreground items of interest rather than the stationary background. In [38], a semi-supervised anomaly detection method based on GAN is provided to gather extra information on the normality distribution of the data in order to overcome the limitation of merely acquiring normality in a single domain. To enhance anomaly detection accuracy and efficiency, a novel multi-frame prediction error-based framework is proposed to reduce the prediction error of a normal frame while increasing the prediction error of an abnormal frame by leveraging the prediction errors of adjacent frames [39]. Considering only local or global normality in the temporal dimension thus are difficult to generalize for various scenarios, Zhao et al. design a two-branch model, Local–Global Normality Network (LGN-Net), to simultaneously learn local and global normality [40]. However, prediction methods mainly focus on the variation of appearance and motion, which is sensitive to occlusion and some changes in these frames, resulting that the normal events may gain a larger predicted error.

#### (3) Transformer-based methods.

Transformer is a deep neural architecture that relies on self-attention mechanisms and was initially developed for natural language processing tasks. In recent years, vision transformer models have been increasingly adopted for video anomaly detection (VAD), achieving performance comparable to or even surpassing that of other network architectures. Recent studies [41–48] indicate a preference for employing transformer models in a hybrid manner to enhance VAD performance, typically by integrating a temporal self-attention network (TSAN) with CNN-based features and/or ViT-extracted representations. For instance, Sharif et al. [41] proposed a multiple instance learning-based generalized architecture termed CNN-ViT-TSAN. In the domain of weakly supervised learning, Ullah et al. [42] introduced the ViT-ARN framework for joint anomaly detection and recognition, which employs a compressed one-class classification model to effectively accommodate resource-constrained devices. Adopting an unsupervised learning paradigm, Hafeez et al. [43] presented a generative transfer learning (GTL) algorithm for VAD, comprising a feature extractor, a generator transformer, and a discriminator transformer. Despite their ability to capture long-term dependencies and achieve superior detection performance, transformer-based methods face significant challenges when deployed on embedded devices with limited hardware resources, primarily due to their high computational and memory demands.

In the proposed method, U-Net is chosen as the base architecture for its balance between representational capacity and computational efficiency, which is critical for real-time video anomaly detection. Unlike TransUNet or SegFormer, which are designed for segmentation tasks and rely heavily on global self-attention, U-Net provides a lightweight and modular structure that allows seamless integration of spatial and channel attention modules without altering the core encoder-decoder design. Furthermore, the skip connection can be particularly effective for preserving fine-grained spatial details required for frame prediction and reconstruction. Therefore, U-Net remains a more suitable backbone for our cascaded prediction-reconstruction architecture.

## 3. Proposed method

In this part, we investigate a cascaded prediction-reconstruction network with hybrid attention for anomaly detection in video surveillance. As shown in Fig 1, the proposed cascaded model is mainly composed of a prediction network and a reconstruction network based on hybrid attention-guided U-Nets. The prediction network can enlarge the error between the original frame and the predicted frame of the anomaly event, meanwhile, the reconstruction network can enhance the reconstruction quality of the normal event. Finally, the advantages of the prediction network and the reconstruction network can be synthesized for recognizing anomaly behaviors.

**Fig 1:**
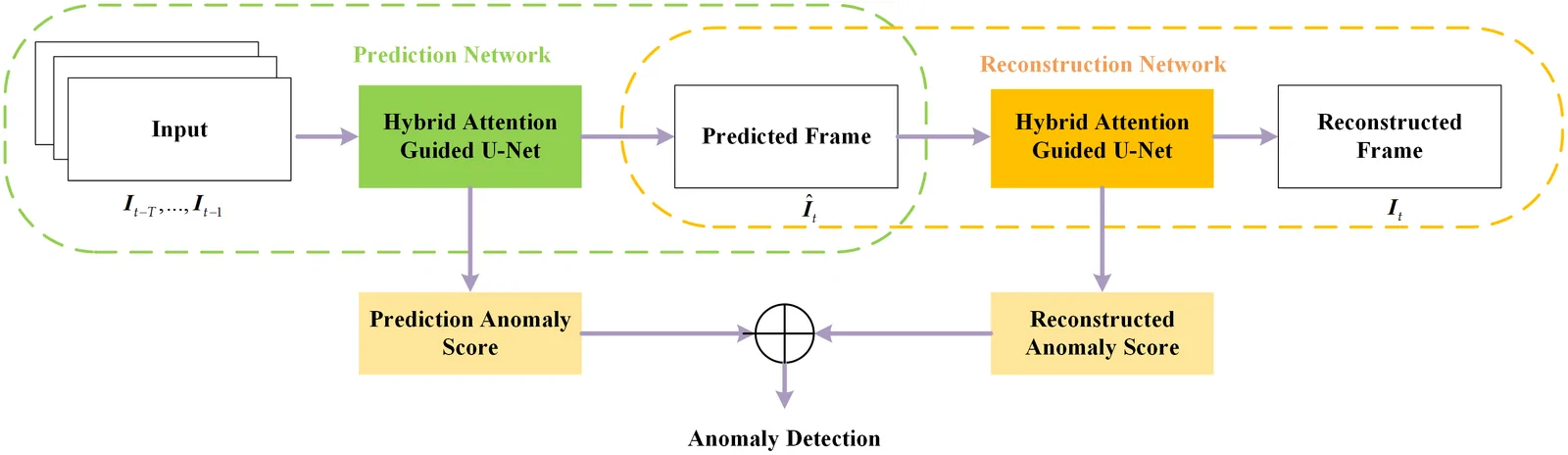
Overall framework of our proposed method.

### 3.1 Network architecture of U-Nets based on hybrid attention

Inspired by [34], by incorporating a shortcut with the same resolution, U-Net can reduce information imbalance and the gradient vanishing issue. In our implementation, we establish our proposed method based on cascaded U-Nets, which mainly includes two stages, frame prediction network and reconstruction network. The cascaded U-Net mainly includes spatial attention module, transformer-based channel attention module, temporal shift module. Specifically, $I_{t-T},...,I_{t-1}$ denotes the input of the U-Nets. In the encoding, feature maps can be obtained by 3×3 convolutional layers and 2×2 max-pooling layers, and the size of feature maps can be represented as H×W×C, which respectively denote height, weight, and the channels of feature maps. As shown in Fig 2, consecutive video frames are the input, the temporal shift module is inserted into the bottleneck layer in the prediction network, the weight of spatial regions and channels conveying background information can be decreased by the addition of the hybrid attention in skip connection, which combines spatial attention module with a transformer-based channel attention module. Different from the prediction network, the frame reconstruction network does not need to pay attention to temporal information, transformer-based channel attention module is added to the bottleneck for focusing the weights of channels, and the spatial attention is added to the skip connection for focusing the weights of spatial regions.

**Fig 2:**
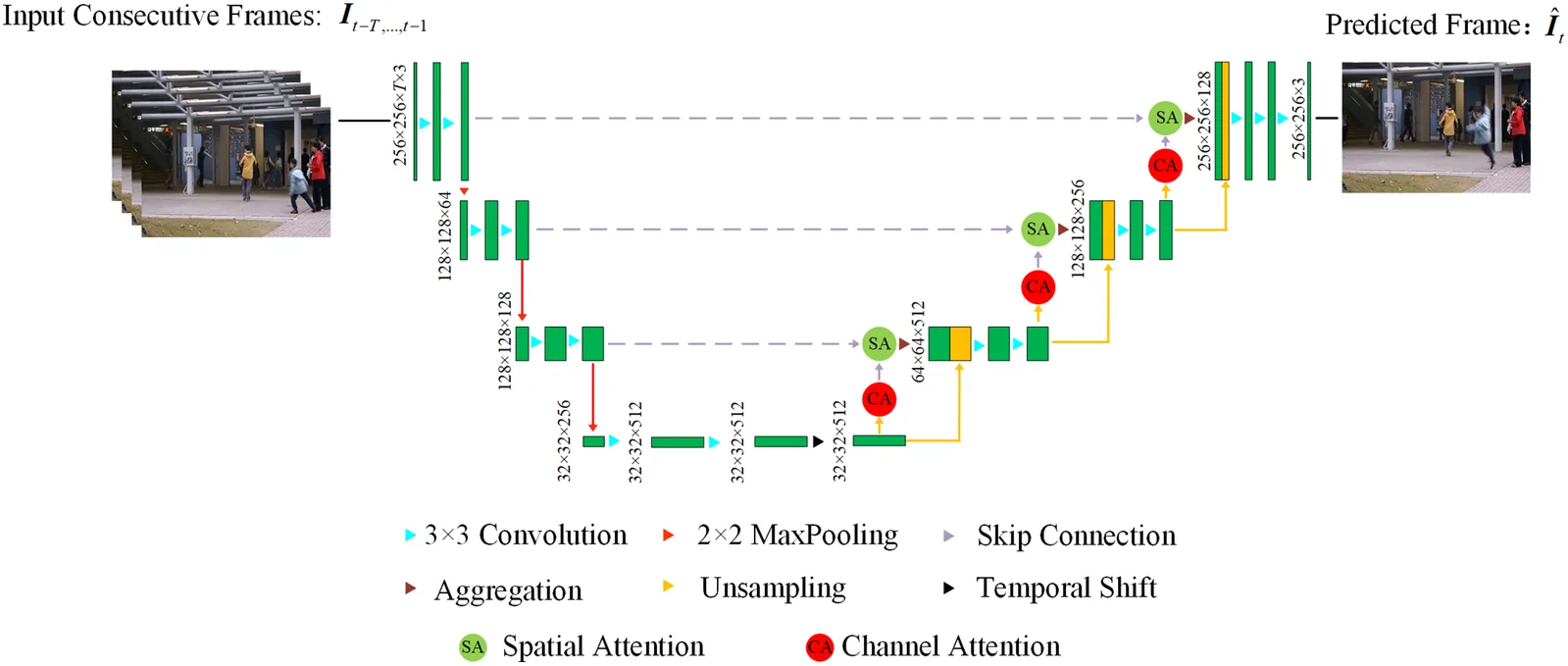
The architecture of the frame prediction network.

Hence, these consecutive video frames can be input into the prediction network *P*, and the predicted frame ${\hat{I}}_{t}$ can be obtained by the *P*. According to the predicted frame ${\hat{I}}_{t}$ and the original frame $I_{t}$, the prediction anomaly detection score ${\hat{S}}_{t}$ can be acquired. the predicted frame ${\hat{I}}_{t}$ is regarded as the input into the reconstruction network *R*, and the reconstructed frame ${\tilde{I}}_{t}$ can be obtained by the *R*.

### 3.2. Transformer-based channel attention module

To better capture complex channel-wise relationships and suppress background-related feature channels, we utilize a transformer-based module to assign the attention weight of the feature channel. The schematic diagram of the channel attention module is shown in Fig 3. First, the average pooling is applied on the feather map X, and the input vector of the channel attention branch can be defined as follows:

**Fig 3:**
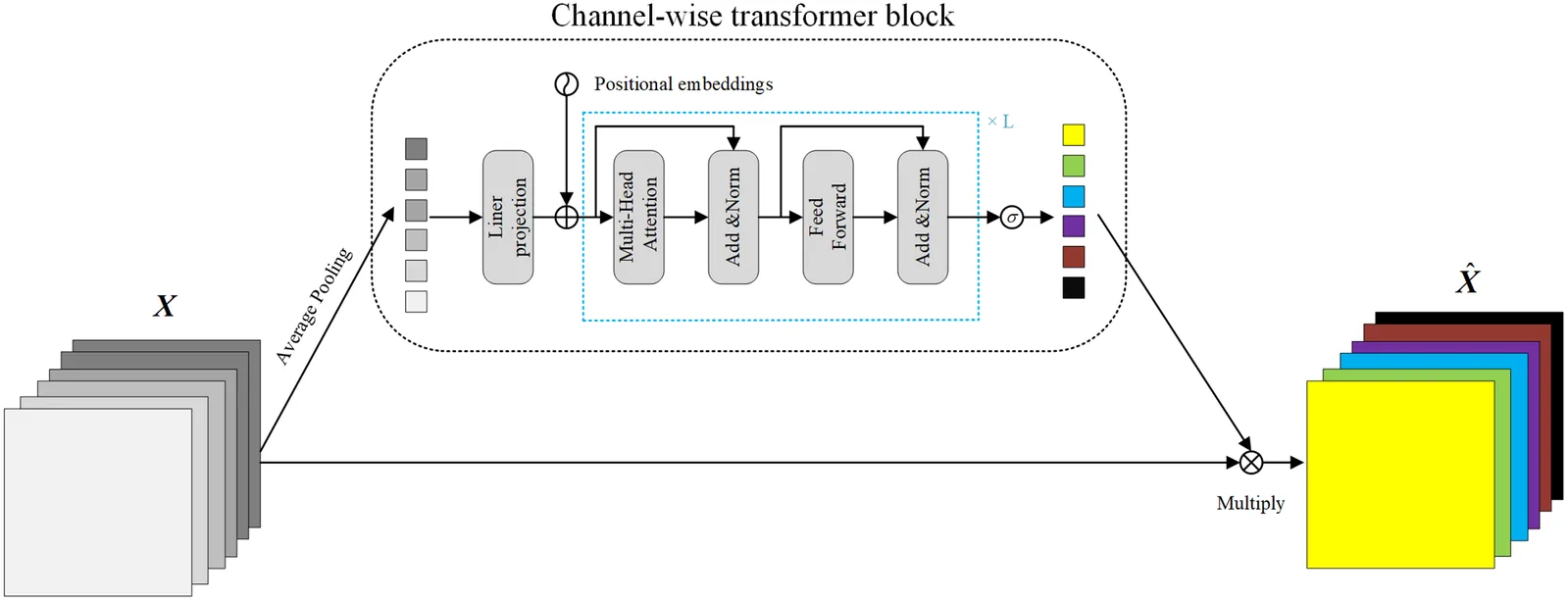
The schematic diagram of the transformer-based channel attention module.


$$\begin{array}{c} A=avgpooling\left(X\right) \end{array}$$ (1)

Next, the input tokens T can be obtained by summing up A and the corresponding position embedding. In the transformer module, the multi-head attention layer simultaneously processes information from multiple representation subspaces across different channels. The output Y of multiple self-attention heads can be defined as follows:


$$\begin{array}{c} Y=Concat\left(Y_{\text{1}},...,Y_{h}\right)W^{O} \end{array}$$ (2)

where $W^{O}\in {\mathbb{R}}^{hd_{v}\times d_{\text{model}}}$ denotes the projections based on multiple self-attention heads, h is the number of self-attention heads. Concretely, the $i_{th}$ self-attention head includes the query matrix $Q_{i}$, the key matrix $K_{i}$, the value matrix $V_{i}$, and the output of a single self-attention head can be represented as follows:


$$\begin{array}{c} \begin{array}{c} {\;Y}_{i}=\sigma \left(\frac{Q_{i}\cdot K_{i}^{T}}{\sqrt{d_{q}}}\right)\cdot V_{i}\mathrm{\backslash hfill}\\ s.t.\;Q_{i}=T\cdot W^{{\;Q}_{i}}\mathrm{\backslash hfill}\\ {\;\;\;K}_{i}=T\cdot W^{{\;K}_{i}}\mathrm{\backslash hfill}\\ {\;\;\;V}_{i}=T\cdot W^{V_{i}}\mathrm{\backslash hfill} \end{array} \end{array}$$ (3)

where σ denotes softmax activation function, $W^{Q_{i}}\in {\mathbb{R}}^{d_{\text{model}}\times d_{q}}$, $W^{K_{i}}\in {\mathbb{R}}^{d_{\text{model}}\times d_{k}}$, $W^{V_{i}}\in {\mathbb{R}}^{d_{\text{model}}\times d_{v}}$ are learnable weight matrices in single self-attention head, and $d_{q}=d_{k}=d_{v}=d_{\text{model}}/h$ is set. Then, the transformer block can be represented as follows:


$$\begin{array}{c} R=norm\left(FNN\left(norm\left(Y+T\right)\right)+norm\left(Y+T\right)\right) \end{array}$$ (4)

in which R denotes the input of the transformer block, FNN denotes the position-wise feed-forward layer and norm denotes the normalization layer. Accordingly, the channels of the feature map X can be weighted by the transformer block, which is represented as follows:


$$\begin{array}{c} \hat{X}=X⊗\sigma \left(R\right) \end{array}$$ (5)

in which σ denotes the softmax activation function, ⊗ is the element-wise multiplication, $\hat{X}$ is the output of the transformer-based channel attention module, which can decrease the weights of the channels representing the background.

### 3.3 Spatial attention module

Further reducing the weights of the feature regions representing the background, we utilize the spatial attention module to focus more on optimizing the regions. The spatial attention module is applied between the features of the encoder and the features of the decoder in the U-Net network. The schematic diagram of the spatial attention module is shown in Fig 4.

**Fig 4:**
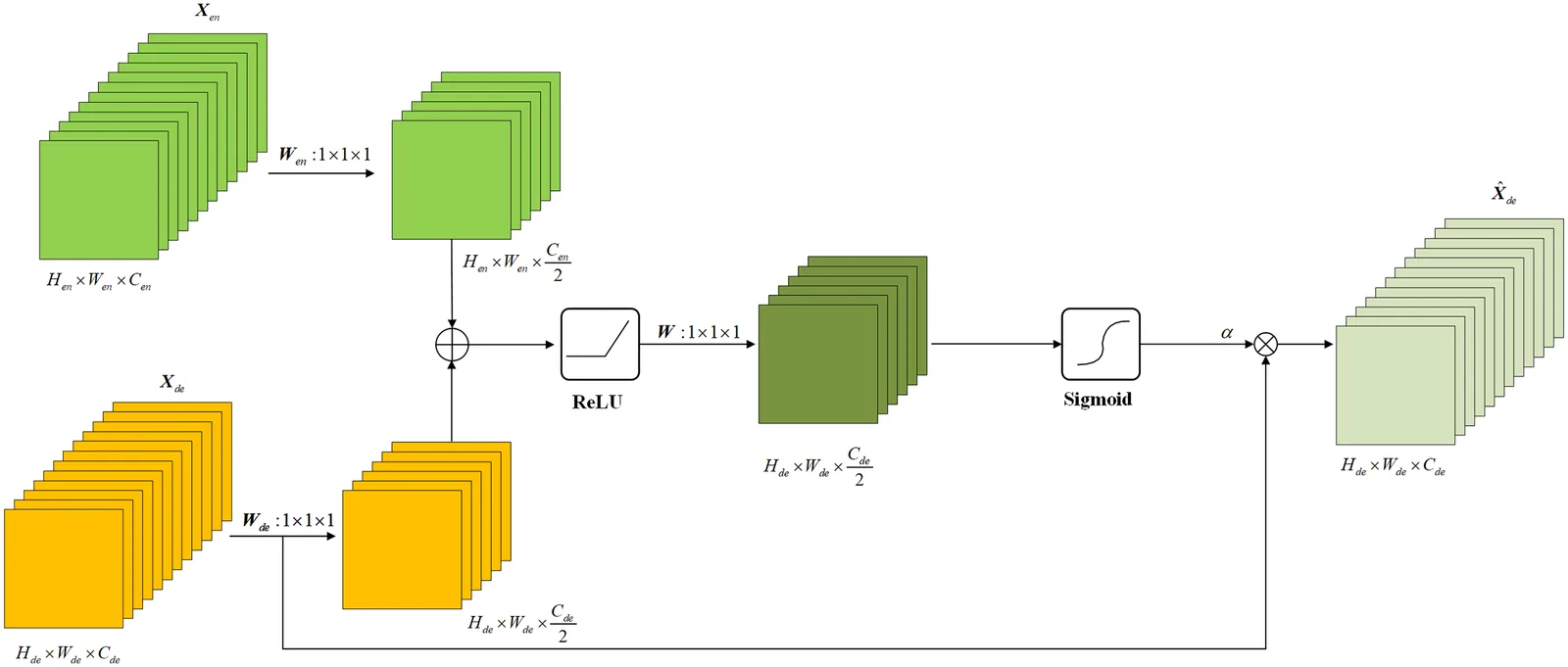
The schematic diagram of the spatial attention module.

Specifically, $X_{en}$ denotes the features of the encoder layer, and the size can be represented as $H_{en}\times W_{en}\times C_{en}$. $X_{de}$ denotes the features of the encoder layer, and the size can be represented as $H_{de}\times W_{de}\times C_{de}$. Since the features of the encoder layer and the features of the encoder layer are connected by the skip connection layer, the number of channels, height, and width of the matrix are the same between $X_{en}$ and $X_{de}$. As such, $X_{en}$ and $X_{de}$ can be combined to compute the attention coefficient, which can be represented as follows:


$$\begin{array}{c} \alpha =\sigma \left(W_{\alpha }•\left(\delta \left(W_{en}•X_{en}+b_{en}\right)+\delta \left(W_{de}•X_{de}+b_{de}\right)\right)+b_{\alpha }\right) \end{array}$$ (6)

where $W_{en}$, $W_{de}$ and $W_{\alpha }$ respectively denote the learned weight matrices, $b_{en}$, $b_{de}$ and $b_{\alpha }$ denote bias matrices, σ and δ respectively denote the sigmoid activation function and ReLU activation function.

Ultimately, the spatial regions of the feature map $X_{en}$ can be weighted by the attention coefficient, and the weights of the spatial regions representing the background can be reduced, which is represented as follows:


$$\begin{array}{c} {\hat{X}}_{en}=\alpha \cdot X_{en} \end{array}$$ (7)

### 3.4 Temporal shift module

To make full use of temporal information, we adopt the temporal shift module in the prediction network to achieve more accurate frame prediction. Different from the traditional 3D CNN method, TSM will move a portion of the channels toward the temporal direction, facilitating information sharing between frames that are neighboring. The feature channels of several different frames can be represented as various colors in each column, as illustrated in Fig 5. Some of the channels are kept and others are transferred to the next frame, the shifted blank channels can be filled with pad zero, additional channels can be removed and truncated.

**Fig 5:**
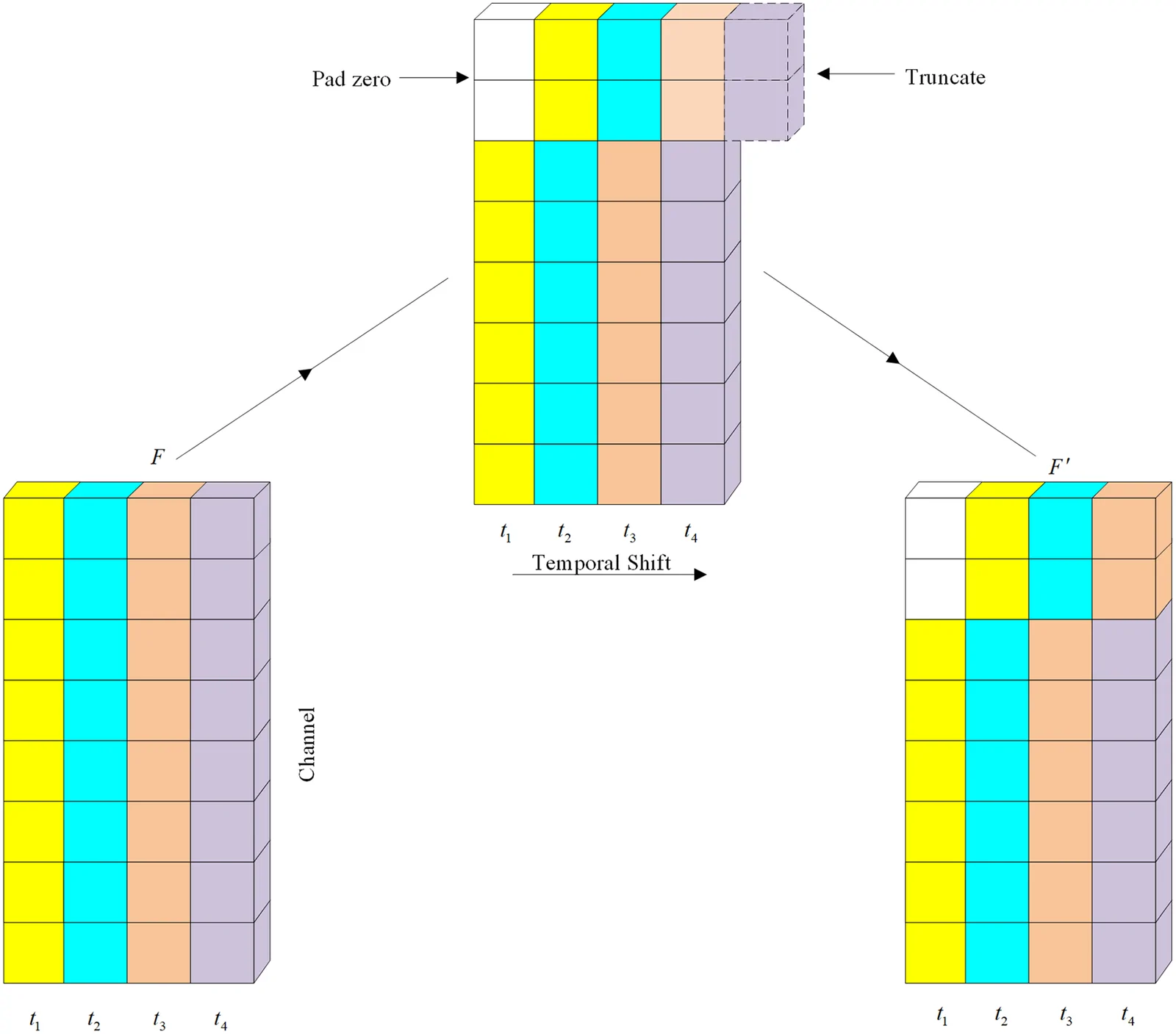
The process of temporal shifting.

Concretely, the temporal shift process can be explained by a 1-D convolution. In detail, $W=\left(w_{\text{1}},w_{\text{2}},w_{\text{3}}\right)$ that kernel size is 3 denotes convolution, for 1-D vector $X=\left(x_{\text{1}},x_{\text{2}},x_{\text{3}}\right)$, the output of convolution can be obtained: $Y=w_{\text{1}}\times x_{\text{1}}+w_{\text{2}}\times x_{\text{2}}+w_{\text{3}}\times x_{\text{3}}$. Then, the 1-D vector X can be shifted to a step size of 1, the shifted 1-D vector can be represented as $X^{\prime }=\left(0,x_{\text{1}},x_{\text{2}}\right)$, and the output of convolution can be obtained: $Y^{\prime }=w_{\text{1}}\times 0+w_{\text{2}}\times x_{\text{1}}+w_{\text{3}}\times x_{\text{2}}$. However, it is worth noting that the channels of features in anomaly behaviors detection are too larger, and large data movement will result in worse efficiency and performance degradation. Consequently, the proportion of the channels can be shifted in the temporal shift module, which can focus on temporal information while bringing down the memory movement cost. Besides, the capacity of 2D CNN for spatial modeling may be harmed by shifting the channels. Consequently, a temporal shift process is combined with a skip connection, and the temporal shift module can easily be integrated into 2D CNN, as shown in Fig 6. Since all of the data in the initial activation is still accessible after a temporal shift via identity mapping, skip connection can solve the impaired spatial feature learning problem.

**Fig 6:**
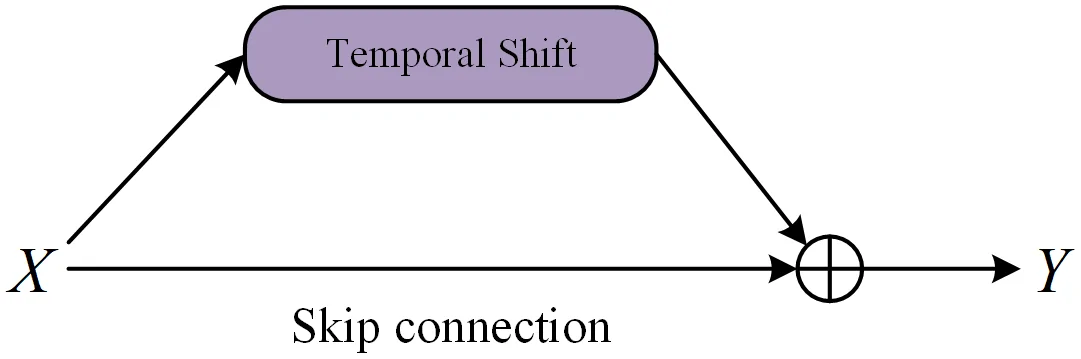
The temporal shift module.

### 3.5 Loss function

To more effectively and completely detect anomalies, designing a loss function is generally vital to achieving convergence and improving performance. In our method, frame prediction and frame reconstruction make up the overall network, and appearance constraints and adversarial constraint can be jointly optimized.

#### (1) Appearance constraints.

To ensure the appearance similarity of the predicted frame and reconstructed frame as similar as possible to the corresponding target frame, the pixel intensity loss and the gradient loss can be adopted. Specifically, the pixel intensity loss based on the l_2_ norm can be represented as follows:


$$\begin{array}{c} \begin{array}{c} L_{int}\left(I_{t},{\hat{I}}_{t},{\tilde{I}}_{t}\right)=L_{int\_p}\left(I_{t},{\hat{I}}_{t}\right)+L_{int\_r}\left(I_{t},{\tilde{I}}_{t}\right)\mathrm{\backslash hfill}\\ \;\;\;\;\;\;\;=\sum_{i,j}{\|I_{t}-{\hat{I}}_{t}\|}_{\text{2}}+\sum_{i,j}{\|I_{t}-{\tilde{I}}_{t}\|}_{\text{2}}\mathrm{\backslash hfill} \end{array} \end{array}$$ (8)

where i is the index of the row, j is the index of the column, ${\hat{I}}_{t}$, ${\tilde{I}}_{t}$, $I_{t}$ are respectively the predicted frame of the prediction network, the reconstructed frame of the reconstruction network, and the real frame. Additionally, based on the horizontal and vertical orientation, the gradient difference can be represented as follows:


$$\begin{array}{c} \Delta_{grad}^{\left[i\right]}\left(I,i,j\right)=\left|I\left(i,j\right)-I\left(i-1,j\right)\right| \end{array}$$ (9)

$$\begin{array}{c} \Delta_{grad}^{\left[j\right]}\left(I,i,j\right)=\left|I\left(i,j\right)-I\left(i,j-1\right)\right| \end{array}$$ (10)

Thus, the gradient loss based on the *l*_*1*_ norm can be represented as follows:


$$\begin{array}{c} \begin{array}{c} L_{grad}\left(I_{t},{\hat{I}}_{t},{\tilde{I}}_{t}\right)\\ =L_{grad\_p}\left(I_{t},{\hat{I}}_{t}\right)+L_{grad\_r}\left(I_{t},{\tilde{I}}_{t}\right)\\ =\sum_{i,j}{\|\Delta_{grad}^{\left[i\right]}\left(I_{t},i,j\right)-\Delta_{grad}^{\left[i\right]}\left({\hat{I}}_{t},i,j\right)\|}_{\text{1}}+\sum_{i,j}{\|\Delta_{grad}^{\left[j\right]}\left(I_{t},i,j\right)-\Delta_{grad}^{\left[j\right]}\left({\hat{I}}_{t},i,j\right)\|}_{\text{1}}\\ +\sum_{i,j}{\|\Delta_{grad}^{\left[i\right]}\left(I_{t},i,j\right)-\Delta_{grad}^{\left[i\right]}\left({\tilde{I}}_{t},i,j\right)\|}_{\text{1}}+\sum_{i,j}{\|\Delta_{grad}^{\left[j\right]}\left(I_{t},i,j\right)-\Delta_{grad}^{\left[j\right]}\left({\tilde{I}}_{t},i,j\right)\|}_{\text{1}} \end{array} \end{array}$$ (11)

Consequently, the appearance loss can be represented as follows:


$$\begin{array}{c} L_{apperance}\left(I_{t},{\hat{I}}_{t},{\tilde{I}}_{t}\right)=\alpha_{int}L_{int}\left(I_{t},{\hat{I}}_{t},{\tilde{I}}_{t}\right)+\alpha_{grad}L_{grad}\left(I_{t},{\hat{I}}_{t},{\tilde{I}}_{t}\right) \end{array}$$ (12)

#### (2) Adversarial constraint.

To enhance the quality of the predicted frame and reconstructed frame, the adversarial constraint is adopted in our proposed method. In detail, the prediction network and the reconstruction network are considered as the generative model *G*, and the discriminative model *D* consists of fully connection layers, which aims to discriminate the frames generated by *G*. Specifically, optimizing *D* needs to fix the weights of *G*, the loss function based on mean square error (MSE) can be represented as follows:


$$\begin{array}{c} \begin{array}{c} L_{adv}^{D}=L_{adv\_p}^{D}\left(I_{t},{\hat{I}}_{t}\right)+L_{adv\_r}^{D}\left(I_{t},{\tilde{I}}_{t}\right)\\ =\sum_{i,j}\frac{\text{1}}{\text{2}}L_{mse}\left(D{\left(I_{t}\right)}_{i,j},1\right)+\sum_{i,j}\frac{\text{1}}{\text{2}}L_{mse}\left(D{\left({\hat{I}}_{t}\right)}_{i,j},0\right)\\ +\sum_{i,j}\frac{\text{1}}{\text{2}}L_{mse}\left(D{\left(I_{t}\right)}_{i,j},1\right)+\sum_{i,j}\frac{\text{1}}{\text{2}}L_{mse}\left(D{\left({\tilde{I}}_{t}\right)}_{i,j},0\right) \end{array} \end{array}$$ (13)

in which $D{\left(I_{t}\right)}_{i,j}$ takes values in {0,1}, and $D{\left({\hat{I}}_{t}\right)}_{i,j}\in \left[0,1\right]$, $D{\left({\tilde{I}}_{t}\right)}_{i,j}\in \left[0,1\right]$. Correspondingly, optimizing *G* needs to fix the weights of *D*, the generative loss can be represented as follows:


$$\begin{array}{c} \begin{array}{c} L_{adv}^{G}\left({\hat{I}}_{t},{\tilde{I}}_{t}\right)\\ =\sum_{i,j}\frac{\text{1}}{\text{2}}L_{mse}\left(D{\left({\hat{I}}_{t}\right)}_{i,j},1\right)+\sum_{i,j}\frac{\text{1}}{\text{2}}L_{mse}\left(D{\left({\tilde{I}}_{t}\right)}_{i,j},1\right) \end{array} \end{array}$$ (14)

To this end, the final loss function with appearance constraints and adversarial constraint can be represented as follows:


$$\begin{array}{c} L=\alpha_{int}L_{int}\left(I_{t},{\hat{I}}_{t},{\tilde{I}}_{t}\right)+\alpha_{grad}L_{grad}\left(I_{t},{\hat{I}}_{t},{\tilde{I}}_{t}\right)+\alpha_{adv}L_{adv}^{G}\left({\hat{I}}_{t},{\tilde{I}}_{t}\right) \end{array}$$ (15)

in which $\alpha_{int}$, $\alpha_{grad}$ and $\alpha_{adv}$ are respectively the coefficient factors of intensity loss, gradient loss, and adversarial loss, which can control the contribution of different loss functions.

### 3.6 Anomaly detection score

Video anomaly detection aims to assign an anomaly score to each frame, reflecting the likelihood of containing anomalous events. While Peak Signal-to-Noise Ratio (PSNR) is commonly used to evaluate frame quality, our method enhances discriminability by prediction network and reconstruction network. The final anomaly score is computed by fusing PSNR-based prediction and reconstruction errors. Specifically, PSNR is defined as:


$$\begin{array}{c} \text{PSNR}\left({\overset{―}{I}}_{t},I_{t}\right)=10log_{10}\frac{[{max}_{{\overset{―}{I}}_{t}}{]}^{\text{2}}}{\frac{\text{1}}{h\times w}\sum_{i=0}^{h-1}\sum_{j}^{w-1}{\left({\overset{―}{I}}_{t}\left(i,j\right)-I_{i}\left(i,j\right)\right)}^{\text{2}}} \end{array}$$ (16)

where ${\overset{―}{I}}_{t}$ denotes the $t_{th}$ generated frame, $I_{t}$ denotes the $t_{th}$ real frame, (i,j) is the index of the frame, h and w are respectively the height and the weight of the frame. Usually, the higher value of PSNR represents better quality of generated frame obtained by the prediction network and reconstruction network. As a result, the anomaly detection score $\hat{S}\left(t\right)$ of the predicted frame generated by the prediction network can be represented as follows:


$$\begin{array}{c} \hat{S}\left(t\right)=\frac{PSNR\left({\hat{I}}_{t},I_{t}\right)-{min}_{i}PSNR\left({\hat{I}}_{i},I_{i}\right)}{{max}_{i}PSNR\left({\hat{I}}_{i},I_{i}\right)-{min}_{i}PSNR\left({\hat{I}}_{i},I_{i}\right)} \end{array}$$ (17)

where ${\hat{I}}_{t}$ denotes the $t_{th}$ predicted frame, i is the index of the frame in the video. Similarly, the anomaly detection score $\tilde{S}\left(t\right)$ of the reconstructed frame generated by the reconstruction network can be represented as follows:


$$\begin{array}{c} \tilde{S}\left(t\right)=\frac{PSNR\left({\tilde{I}}_{t},I_{t}\right)-{min}_{i}\;PSNR\left({\tilde{I}}_{i},I_{i}\right)}{{max}_{i}\;PSNR\left({\tilde{I}}_{i},I_{i}\right)-{min}_{i}\;PSNR\left({\tilde{I}}_{i},I_{i}\right)} \end{array}$$ (18)

where ${\tilde{I}}_{t}$ denotes the $t_{th}$ reconstructed frame. In this way, the overall anomaly score is the combination of the predicted anomaly score and the reconstructed anomaly score, which can be represented as follows:


$$\begin{array}{c} S_{all}\left(t\right)=\mu \hat{S}\left(t\right)+\upsilon \tilde{S}\left(t\right) \end{array}$$ (19)

where μ, ν are the impact factors for controlling the contribution of the corresponding score, which is adjustable according to the significance of the predicted anomaly score and the reconstruction score. Eventually, the threshold can be set to recognize anomaly behaviors, higher anomaly scores usually denote anomalies, and lower anomaly scores denote regularities.

## 4. Experiments

We evaluate our method on three public datasets to validate its anomaly detection performance. First, we describe the benchmark datasets and implementation details. Then, we analyze the impact of our key components: transformer-based channel attention module, spatial attention module, and temporal shift module. Finally, we compare our proposed cascaded prediction-reconstruction network with state-of-the-art methods. Experimental details are provided in the following sections.

### 4.1 Datasets and evaluation metrics

#### *(1)* Datasets.

We conduct experiments on four public benchmarks.

***UCSD Pedestrian Dataset:*** Mahadevan *et al.* [49] created the UCSD Pedestrian Dataset of two scenes, which consists of two sub datasets named Ped1 dataset and Ped2 dataset. The Ped1 dataset includes 70 grayscale videos in total, of which 34 are training videos and 36 are testing videos with a resolution of 158 × 238 pixels. The Ped2 dataset includes 28 grayscale videos in total, of which 16 are training videos and 12 are testing videos with a resolution of 320 × 240 pixels. The emergence of vehicles including bicycles, trucks, and cars is the primary cause of the anomalous events.

***CUHK Avenue Dataset:*** Lu *et al.* [50] created the CUHK Avenue Dataset of a single scene in front of a subway station. The dataset includes 37 RGB videos in total, of which 16 are training videos and 21 are testing videos with a resolution of 640 × 360 pixels and a frame rate of 25 frames per second. The normal events among them include people walking on the street, but the abnormal ones mostly involve throwing things, straying, sprinting, or walking in the opposite way, etc..

***ShanghaiTech Dataset:*** Luo *et al.* [51] created the ShanghaiTech Dataset of 13 single scenes captured from the campus of ShanghaiTech University. The dataset includes 437 RGB videos in total, of which 330 are training videos and 107 are testing videos with a resolution of 640 × 360 pixels and a frame rate of 25 frames per second. The abnormal events mostly involve riding bikes, fighting, and vehicles. Compared with existing benchmarks, the ShanghaiTech dataset is a more challenging dataset..

***UCF-Crime Dataset:*** UCF-Crime [52] is an extensive collection of real-world videos recorded by surveillance cameras, which contains 1900 videos and includes 13 distinct types of anomalies, such as shootings and abuse. Each video is annotated with a binary label, where 0 indicates normal and 1 indicates abnormal. The dataset is divided into a training set comprising 1610 videos, with 800 labeled as normal and 810 as abnormal, and a testing set consisting of 290 videos, of which 150 are normal and 140 are abnormal..

#### (2) Implementation details.

We implemented our proposed cascaded U-Nets using PyTorch on a server with one RTX 2080Ti GPU. The model was trained for 50 epochs with Adam based on stochastic gradient descent method. In the training processing, all frames were resized to 256 × 256, the initial learning rate for the generative model *G* and the discriminative model *D* was respectively set to 0.0002 and 0.00002, and batch size of 4 was adopted for all datasets. The length of consecutive video frames for the prediction network was set to T=4. Based on the data characteristics of each dataset, we set the hyperparameter μ in equation 19 differently: 0.5 for Ped1 and Ped2, 0.3 for Avenue, 0.1 for ShanghaiTech, and 0.3 for UCF-crime. According to the experimental results and experience, and the coefficient factors of intensity loss, gradient loss, and adversarial loss were respectively set to $\alpha_{int}=1$, $\alpha_{grad}=1$ and $\alpha_{adv}=0.05$ in equation (19) for all datasets.

In addition, to ensure the fairness and reproducibility of performance comparisons, we strictly follow a unified evaluation protocol across all datasets and competing methods, as recommended by recent methodological studies such as [53]. Specifically, we impose the following consistency constraints. Fixed data split: all compared methods are trained on the official training set and tested on the official testing set of each benchmark. Evaluation metric alignment: frame-level AUC is adopted as the universal metric. Anomaly scores for each frame are computed using the same PSNR-based normalization. The ROC curve is generated by varying the decision threshold from 0 to 1 without any dataset-specific tuning. For cases where results could not be reproduced, or where reproduced results exhibited lower performance, we adopted the results reported in the original publications.

#### (3) Evaluation metrics.

The extent of anomalies for each frame is determined by using an anomaly detection score based on equation (18) during the inference phase. It is classified as anomalous or otherwise normal if the anomaly score of the predicted and reconstructed frame exceeds the judgment threshold. In line with other research [34,54], we calculate the average area under the receiver operating characteristic (AUC) by computing the area under the Receiver Operating Characteristic (ROC). The threshold is changed to produce the ROC curve. A higher AUC value indicates a better capacity to detect abnormalities.

### 4.2 Performance comparison with existing methods

In this section, to validate the effectiveness and generalization ability of our cascaded U-Nets for anomaly detection, we compare our method with other existing state-of-the-art methods including traditional methods, deep reconstruction methods, deep prediction methods and deep hybrid methods.

As shown in Table 1, the AUC comparisons of different methods on the Avenue dataset, the Ped1 dataset, the Ped2 dataset, and the ShanghaiTech dataset are listed. Compared with traditional approaches based on hand-crafted features and one-class classifiers, our method demonstrates clear improvements in AUC across all datasets, highlighting the advantage of deep learning-based feature learning. Among reconstruction-based and prediction-based deep learning methods, our approach also performs favorably, which we attribute to the combined strengths of the prediction and reconstruction networks in our architecture. The top-performing methods on individual datasets include HF2-VAD (99.3% on Ped2), Attention-Driven Loss (94.2% on Ped1), and HSC (93.7% on Avenue, 83.4% on ShanghaiTech). While these methods achieve high AUC in specific settings, they often come with limitations such as sensitivity to motion speed, restriction to human-related scenes, or dependence on higher-resolution data. In contrast, our method maintains strong overall performance without these constraints, demonstrating robust generalization across different anomaly detection scenarios. Overall, the results confirm that our method attains competitive ranking among state-of-the-art techniques while avoiding several common practical limitations, as further detailed in Table 1.

**Table 1:** AUC comparisons of different methods on the Avenue, Ped1, Ped2 and ShanghaiTech dataset.

Types	Methods	Year	Ped2	Ped1	Avenue	ShanghaiTech
Traditional methods	MPPCA [55]	2009	69.3%	59.0%	-	-
	MDT [49]	2010	82.9%	81.8%	-	-
	Unmasking [56]	2017	82.2%	68.4%	80.6%	-
	LSH [13]	2023	88.8%	-	86.5%	72.1%
Depp Reconstruction Methods	Conv-AE [23]	2016	85.0%	75.0%	80.0%	60.9%
	Conv-LSTM-AE [30]	2017	88.1%	75.5%	77.0%	-
	Stacked RNN [51]	2017	92.2%	-	81.7%	68.0%
	Autoregression Conv-AE [57]	2019	95.4%	-	-	72.5%
	MemAE [58]	2019	94.1%		84.9%	72.8%
	MAND [54]	2020	97.0%	-	88.5%	70.5%
	DPU [59]	2021	96.9%	85.1%	89.5%	73.8%
	Hybrid Attention [36]	2023	95.8%	85.2%	84.9%	71.4%
	Influence-Aware Attention [29]	2022	92.9%	**94.2%**	80.5%	80.3%
Deep Prediction Methods	Baseline [34]	2018	95.4%	83.1%	84.9%	72.8%
	STAE [31]	2017	92.3%	91.2%	80.9%	-
	Attention-Driven Loss [37]	2020	96.0%	83.9%	86.0%	-
	F^2^PN [60]	2022	96.2%	84.3%	85.7%	73.0%
	Multispace [38]	2021	95.4%	-	86.8%	73.6%
	Bi-directional [61]	2022	98.3%		90.3%	78.1%
	ROADMAP [62]	2022	96.3%	83.4%	88.3%	76.6%
	VADNet [24]	2023	96.8%	-	87.3%	75.2%
Deep Hybrid Methods	Integrating Model [63]	2020	96.2%	82.6%	83.7%	71.5%
	HF^2^-VAD [52]	2021	**99.3%**	-	91.1%	76.2%
	HSC [32]	2023	98.1%		**93.7%**	**83.4%**
	**Proposed method**		95.6%	84.1%	**87.6%**	75.1%

In addition, we have further evaluated our proposed method on UCF-Crime dataset to demonstrate the superiority of the proposed network. As shown in Table 2, the AUC comparisons of different methods on the UCF-Crime dataset are listed. Compared with the SACR [64] and Integrating Model [63], the AUC value of the proposed method is 73.5%, achieving an improvement of 0.8% and 0.3% respectively. These results can demonstrate the superiority of the proposed cascaded method in mining complete anomalies.

**Table 2:** AUC comparisons of different methods on the UCF-Crime dataset.

Method	AUC
SACR [64]	72.7%
Integrating Model [63]	73.2%
Proposed method	73.5%

In order to further evaluate the detection capacity of various methods, we compare three metrics, including the average score of all normal frames, the average score of all anomaly frames, and the score gap between the average scores of all normal frames and all anomaly frames, on three public datasets for HAMC [36], MNAD [54], and the proposed method. The comparisons of three measures for HAMC, MNAD, and the proposed method are illustrated in Fig 7. As shown in Fig 7, the orange column denotes the average scores of normal frames, the green column denotes the average scores of anomaly frames, and the purple column denotes the gaps between the average scores of all normal frames and all anomaly frames. For the testing set in the Ped2 dataset, the Ped1 dataset, the CUHK Avenue dataset, and the ShanghaiTech dataset, the score gaps obtained by our proposed method are respectively 0.469, 0.264, 0.308, and 0.184, which are obviously bigger than those obtained by HAMC and MNAD. Usually, a larger score gap denotes easier distinction. Therefore, the comparison of these three measures demonstrates that the proposed method has a greater ability to discriminate between normal events and abnormal events.

**Fig 7:**
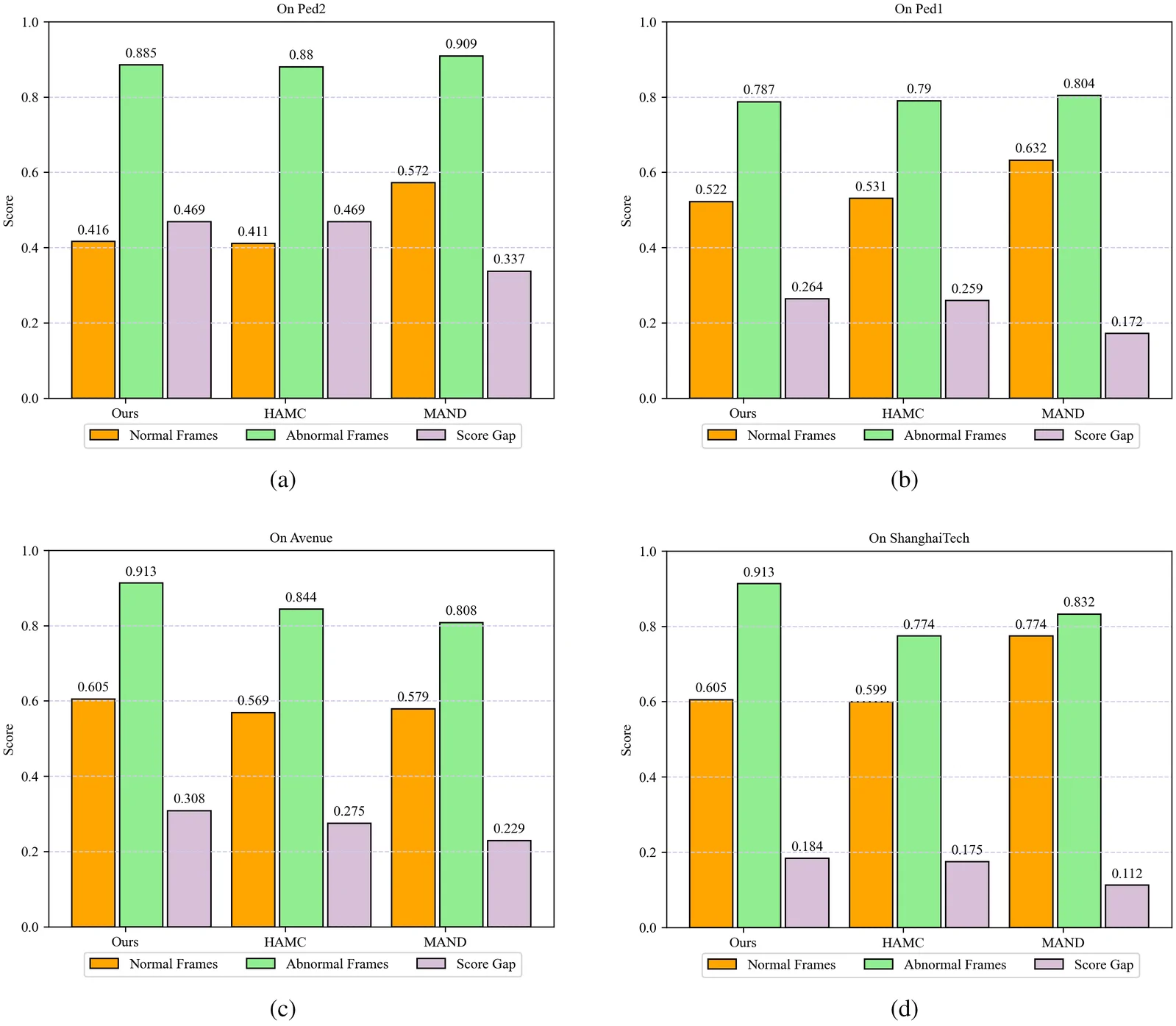
Average scores of normal and abnormal frames and the score gaps for HAMC, MNAD, and the proposed method. (a) The scores on the test set of the Ped2 dataset. (b) The scores on the test set of the Ped1 dataset. (c) The scores on the test set of the Avenue dataset. (d) The scores on the test set of the ShanghaiTech dataset.

### 4.3 Cross-scene anomaly detection

In this section, we perform cross-database experiments to validate that our framework has unique cross-scene application capability. We compare the cross-scene application performances considering two pairs of grayscale datasets, namely Ped1 → Ped2 and Ped2 → Ped1. Ped1 → Ped2 denotes that our proposed framework is trained on the Ped1 dataset and tested on the Ped2 dataset. Correspondingly, Ped2 → Ped1 denotes that our proposed framework is trained on the Ped2 dataset and tested on the Ped1 dataset. In revised manuscript, we add the AUC comparisons of HSC on cross scenarios. Table 3 shows the AUC comparisons using different anomaly detection methods on crossed grayscale scenarios. For Ped1 → Ped2, the AUC value of our proposed method can reach 90.9%, outperforming MAND by a margin of 2.1%, HAMC by a margin of 0.6% and HSC by a margin of 0.4%. In turn, for Ped1 → Ped2, the AUC value of our proposed method can reach 76.9%, outperforming MAND by a significant margin of 10.1%, HAMC by a margin of 7.5% and HSC by a margin of 1.3%. Remarkably, our proposed methods trained on the Ped2 dataset and tested on the Ped1 dataset can achieve a significant improvement. Additionally, we also compare the cross-scene application performances with Background-Agnostic [65] considering two pairs from RGB datasets to grayscale dataset, namely ShanghaiTech→Ped2 and Avenue→Ped2. Table 4 shows the AUC comparison using different anomaly detection on crossed RGB and grayscale scenarios. For ShanghaiTech→Ped2, the AUC value of our proposed method can reach 90.9%, outperforming Background-Agnostic by a margin of 0.3% and HSC by a margin of 0.6%. Moreover, for Avenue→Ped2, the AUC value of our proposed method can reach 89.3%, outperforming Background-Agnostic by a margin of 2.3% and HSC by a margin of 0.7%. It can be demonstrated that our proposed method has a significant capacity to detect anomalous occasions on cross-scene applications since numerous state-of-the-art methods [32,36,38,54] trained on the target dataset do not perform as well as our proposed method. Furthermore, despite the fact that three public datasets are different lengths and different image channels, our proposed cascaded attention U-Nets can achieve competitive results, which can further illustrate the robustness of the proposed method.

**Table 3:** AUC comparisons using different anomaly detection methods on cross grayscale scenarios.

Method	Ped1 → Ped2	Ped2 → Ped1
MAND [54]	88.8%	66.8%
HAMC [36]	90.3%	69.4%
HSC [32]	90.5%	75.6%
**Proposed method**	**90.9%**	**76.9%**

**Table 4:** AUC comparisons using different anomaly detection methods on cross RGB and grayscale scenarios.

Method	ShanghaiTech→Ped2	Avenue→Ped2
Background-Agnostic [65]	90.6%	87.0%
HSC [32]	90.3%	88.6%
Proposed method	**90.9%**	**89.3%**

### 4.4 Visualization

In order to further demonstrate the effectiveness of the cascaded attention U-Nets, several anomaly video samples are selected for testing, and the corresponding PSNR values are computed for reflecting anomaly frames and normal frames. Fig 8(a) shows the PSNR curve of the test video sample in the Ped1 dataset. From Fig 8(a), the PSNR values of the first 80 frames are obviously larger than those of the last 80 frames. It is worth noting that the PSNR values suddenly decrease from the 80th frame to the 90th frame, which represents the anomaly event that person begins riding into the sidewalk, and then the PSNR values keep lower, which represents that a person keeps riding on the sidewalk. Fig 8(b) shows the PSNR curve of the test video sample in the Ped1 dataset, which is similar to that in the Ped2 dataset. Fig 8(c) shows the PSNR curve of the test video sample in the Avenue dataset. From Fig 8(c), it is clear that the PSNR values of the first 290 frames, those from the 340th frames to the 580th frames, and those from the 620th frames to the 920th frames are all higher than those from the 290th frames to the 340th frames and those from the 580th frames to the 620th frames. It is worth noting that the PSNR values from the 290th frames to the 340th frames and those from the 580th frames to the 620th frames suddenly decrease and increase, which represents the anomaly event that a person suddenly begins running in the subway station. Fig 8(d) shows the PSNR curve of the test video sample in the ShanghaiTech dataset. From Fig 8(d), it is obvious that the PSNR values of the first 80 frames and those from the 145th frames to the 180th frames are higher than those from the 80th frames to the 145th frames. It is worth noting that the PSNR values from the 80th frames to the 145th frames suddenly decrease, which represents the anomaly event that a person suddenly begins riding on the sidewalk. As we can see from Fig 8, the normal events, such as walking, usually correspond to higher PSNR values, and the anomaly events correspond to lower PSNR values, which can further indicate that the attention cascaded U-Nets is able to recognize anomalous behaviors in videos.

**Fig 8:**
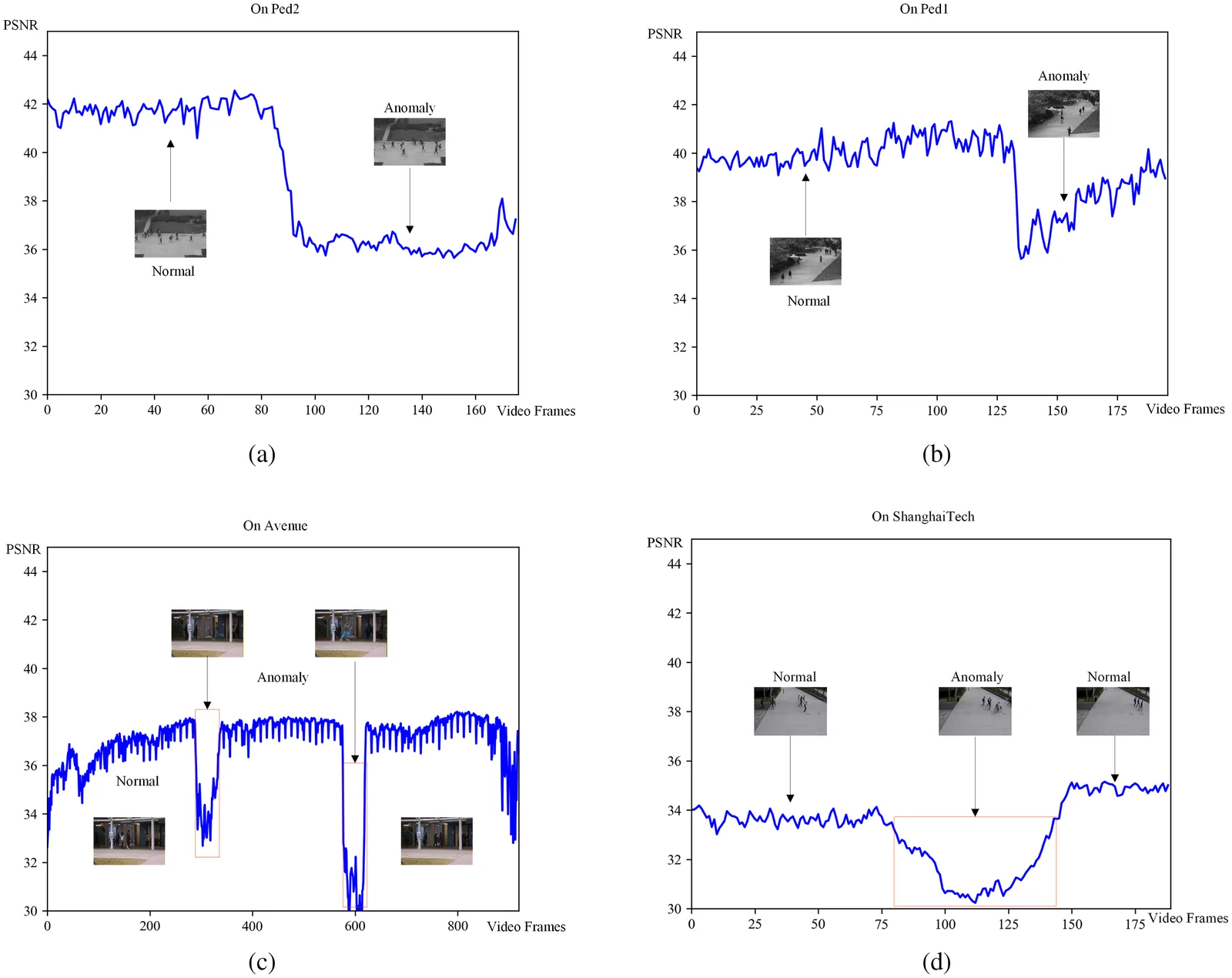
PSRN curves of anomaly video samples. (a) The PSRN curve on the test set of the Ped2 dataset. (b) The PSRN curve on the test set of the Ped1 dataset. (c) The PSRN curve on the test set of the Avenue dataset. (d) The PSRN curve on the test set of the ShanghaiTech dataset.

### 4.5 Ablation studies

To further verify the effectiveness of each component and investigate the influence of impact factors, ablation studies can be performed on public datasets.

#### (1) Impact of different components.

*T*o further demonstrate the influence of different components in our method, with aspect to spatial attention module, temporal shift module, and transformer-based channel attention module, we evaluate the performance using different modules on the Avenue dataset. The ROC curves using different modules on the Avenue dataset are compared in Fig 9. In detail, the yellow curve denotes the baseline module. The pink curve denotes that the baseline module is combined with the spatial attention module. The red curve denotes that the baseline module is combined with the spatial attention module and temporal shift module. The black curve denotes that the baseline module is combined with the spatial attention module, temporal shift module, and channel attention module. As shown in Fig 9, the AUC values using different modules are compared on the Avenue dataset. From the Fig 9, the AUC value of the baseline module based on cascaded U-Nets is 85.1%. It is worth noting that the AUC values of the baseline module with spatial attention module, the baseline module with spatial attention module and temporal shift module, the baseline module with the spatial attention module, temporal shift module, and channel attention module can achieve 86.1%, 87.0% and 87.6%, outperforming the baseline module by a margin of 1.0%, 1.9%, and 2.5%, respectively. The gain of 1% can verify that the introduction of the spatial attention module can improve the ability to increase the weights of targets and decrease the weights of background in spatial regions. The gain of 1.9% can prove that the insertion of a temporal shift module can focus on temporal information, which is important for video anomaly detection. The gain of 2.5% can illustrate that the addition of a transformer-based channel attention module can improve the ability to increase the weights of target feature channels and decrease the weights of background feature channels. The results in Fig 9 indicate that the incorporation of spatial attention module, temporal shift module, and channel attention module play a significant role in our proposed method, and further improve the anomaly detection performance.

**Fig 9:**
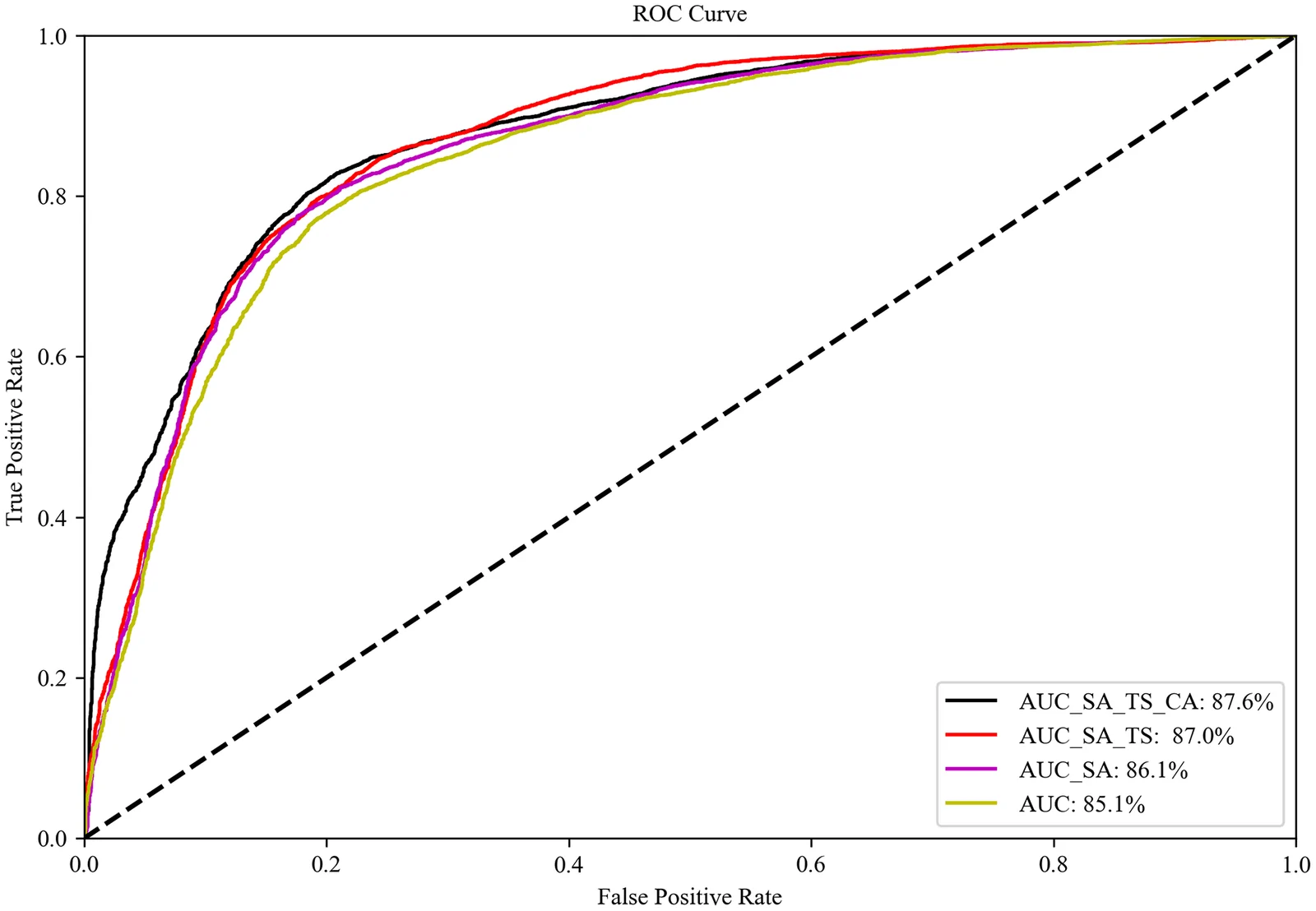
The ROC curve comparisons using different modules on the Avenue dataset.

#### (2) Impact of different scores.

To further demonstrate the effectiveness of the combination of the prediction score and reconstruction score, we evaluate the performance using different anomaly scores on the Avenue dataset, following the study [66]. As shown in Fig 10, the ROC curves and AUC values using different anomaly scores are compared on the Avenue dataset. From Fig 10, the orange curve denotes the module based on prediction score, and the AUC value is 85.1%. Correspondingly, the green curve denotes the module based on reconstruction score, and the AUC value is 87.0%. It is worth noting that the AUC value based on the reconstruction score is higher than that based on the prediction score, which can demonstrate that cascaded U-Nets can improve detection performance. In addition, the purple curve denotes the module with the combination of the prediction score and reconstruction score. It is obvious that the AUC value of the combination of the prediction score and reconstruction score is higher, which can further improve generalization ability.

**Fig 10:**
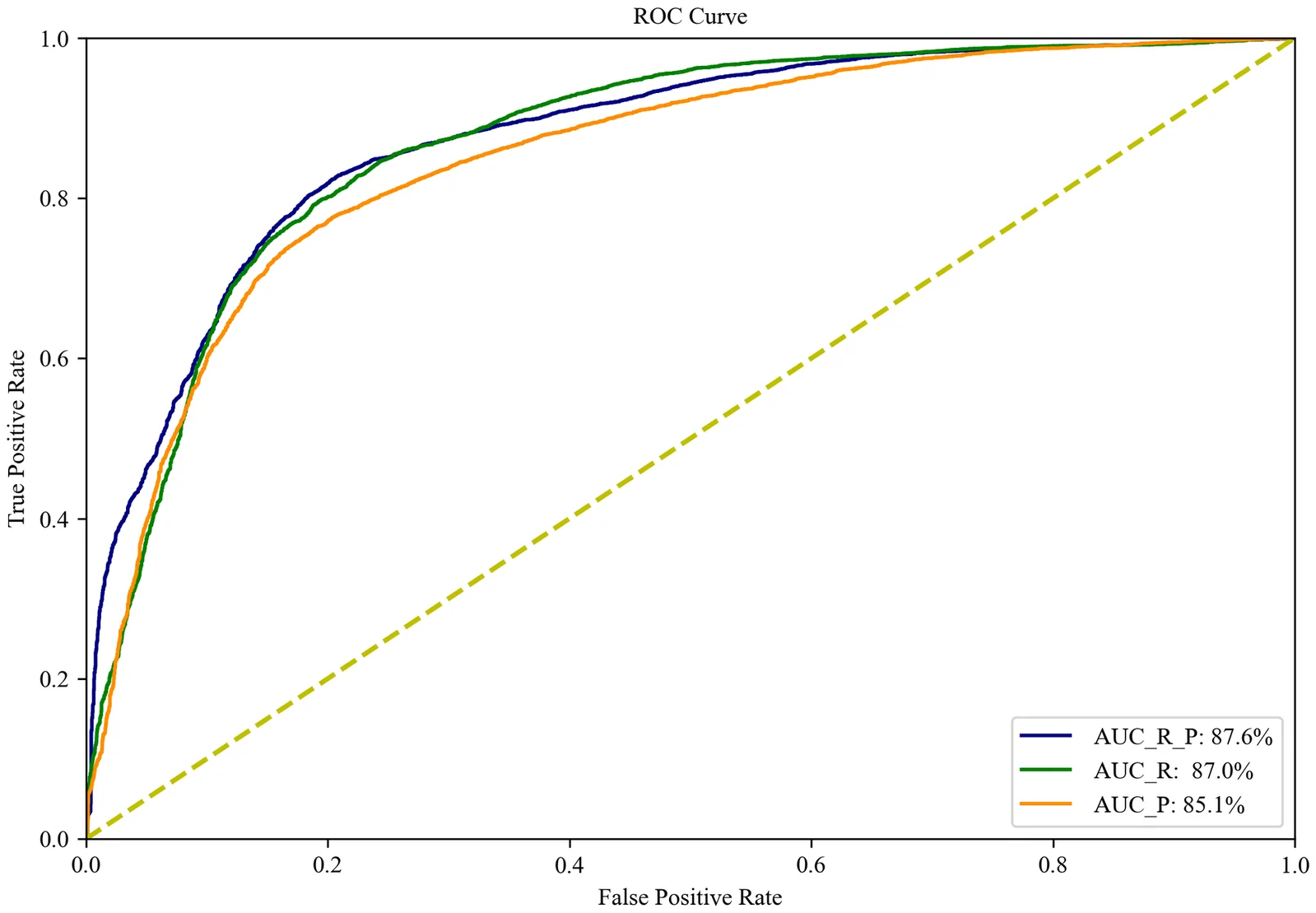
The ROC curve comparisons using different anomaly scores on the Avenue dataset.

In addition, to quantitatively validate the noise robustness of the proposed hybrid framework, we conduct experiments on the Avenue dataset with noise, following the study [67]. We compare three settings: pure prediction network, pure reconstruction network, and our cascaded prediction-reconstruction framework. Gaussian noise with standard deviations σ=10 is added to test videos to simulate real-world interference. Experimental results demonstrate that the pure prediction method suffers severe performance degradation under noise, with AUC drops of 3.8%. The pure reconstruction method is more stable, with AUC drops of 1.7%. In contrast, our hybrid framework maintains strong performance, with AUC drops limited to 1.2%. These quantitative results clearly show that the proposed cascaded structure effectively improves noise robustness by integrating prediction and reconstruction. This complementary behavior is analogous to robust state estimation systems, such as mobile robot localization, which fuse multiple complementary information sources to maintain reliability under uncertain and noisy conditions. The quantitative analysis validates that our hybrid framework achieves stronger noise robustness than single prediction-based or reconstruction-based baselines.

#### (3) Impact of the number of shifted frames.

To determine the optimal frame shift proportion, we conducted experiments on the Avenue dataset following the methodology in [68]. Within the temporal shift module, the shift proportion serves as a critical hyperparameter governing the extent of temporal dependencies the model can capture; specifically, it denotes the proportion of shifted frames relative to the total input frames. Table 5 presents a comparative analysis of the trade-off between temporal delay and detection performance across various shift proportions. As illustrated, an excessively high shift proportion severely impairs the network’s spatial modeling capability, resulting in degraded overall performance. Conversely, an excessively low shift proportion yields insufficient temporal correlation features, which similarly compromises performance. Therefore, by setting the shift proportion to 1/4, this study achieves superior recognition accuracy with only negligible computational overhead.

**Table 5:** Comparisons with different shift proportions on the Avenue dataset.

Shift proportion	0	1/8	1/4	1/2	1
Latency overhead	0%	3%	4.3%	8%	13.8%
AUC	83.5%	86.9%	87.6%	86.3%	84.2%

### 4.6 Robustness analysis

To verify the performance under different levels of occlusion and illumination variations, we conducted randomized validation at UCSD, CUHK Avenue, and ShanghaiTech to simulate occlusion. Table 6 shows the performance comparison under different randomized mask rates. The performance degradation of our model was controlled to be within a small range (< 2%), effectively validating the effectiveness of our method under different levels of occlusion.

**Table 6:** Comparisons with different mask rates on the dataset.

Mask Rates	0	5%	10%
UCSD Ped2	95.6%	94.9%	94.0%
Avenue	87.6%	87.0%	86.4%
ShanghaiTech	75.1%	74.3%	73.9%

### 4.7 Running time

We empirically study the computing complexity of the proposed method on the video dataset UCSD Ped2 with an NVIDIA GeForce 2080 Ti graphics card. The model parameters of our method are 24 million, and the proposed method averagely takes 0.025 seconds for video anomaly detection of one frame (i.e., 40 fps). Here we also compare the computational time of several previous work with our method, which is tested on USCD Ped2 dataset with the same device. The results are illustrated in Table 7. We can see that our approach runs faster. The experimental results mean that our proposed method can be applied for real-time detection.

**Table 7:** Real-time performance on Ped2.

Method	Computational Time (frames per second)
Unmasking [56]	20
Baseline [34]	32
Integrating Model [63]	30
**Our method**	**40**

## 5. Conclusion

The goal of this research is to precisely identify anomalous video events. In particular, we propose a cascaded U-Nets for video anomaly detection which can completely concentrate on the benefit of the frame prediction network and the frame reconstruction network for discriminating anomalies from regular occurrences. Additionally, the temporal shift module is considered an efficient temporal feature extraction to focus on acquiring temporal information. Moreover, the spatial attention module and the transformer-based channel attention module are incorporated to decrease the weight of the background feature in the spatial region and channel region. Extensive experimental results indicate that the proposed cascaded U-Nets based on hybrid attention can achieve remarkable performance. Meanwhile, ablation studies verify that the spatial attention module, the temporal shift module, and the channel attention module can enhance the ability to detect anomaly events. In the future, we would like to investigate how to improve the performance of anomaly detection on cross scenarios.
